# Role of atmospheric resonance and land–atmosphere feedbacks as a precursor to the June 2021 Pacific Northwest Heat Dome event

**DOI:** 10.1073/pnas.2315330121

**Published:** 2024-01-16

**Authors:** Xueke Li, Michael E. Mann, Michael F. Wehner, Stefan Rahmstorf, Stefan Petri, Shannon Christiansen, Judit Carrillo

**Affiliations:** ^a^Department of Earth & Environmental Science, University of Pennsylvania, Philadelphia, PA 19104; ^b^Applied Mathematics and Computational Research Division, Lawrence Berkeley National Laboratory, Berkeley, CA 94720; ^c^Earth System Analysis, Potsdam Institute for Climate Impact Research, Potsdam D-14412, Germany; ^d^Institute of Physics and Astronomy, University of Potsdam, Potsdam 14476, Germany

**Keywords:** climate change, heatwave, atmospheric dynamics, planetary waves, soil moisture

## Abstract

We demonstrate an indirect, rather than direct, role of quasi-resonant amplification of planetary waves in a summer weather extreme. We find that there was an interplay between a persistent, amplified large-scale atmospheric circulation state and soil moisture feedbacks as a precursor for the June 2021 Pacific Northwest “Heat Dome” event. An extended resonant planetary wave configuration prior to the event created an antecedent soil moisture deficit that amplified lower atmospheric warming through strong nonlinear soil moisture feedbacks, favoring this unprecedented heat event.

## Significance

Recent decades have witnessed unprecedented heat waves with severe repercussions for human society. However, a satisfactory explanation of the extremity of some of these events has remained elusive. Here, we demonstrate a combination of factors that contributed to the singularly anomalous Pacific Northwest Heat Dome event of summer 2021, involving the phenomenon of resonant planetary wave amplification—not well represented in state-of-the-art climate models—which interacted with land surface feedbacks to catalyze the extreme heat event. Neglecting such preconditioning feedback mechanisms in climate model analyses could potentially cause underestimates in the future likelihood or severity of extreme continental heat waves. Our findings hold the potential for more skillful predictions of low-probability yet impactful weather extremes that can have devastating consequences.

Heat stress is among the greatest threats to human health posed by anthropogenic climate change (1, 2). The unusual timing, severity, and frequency of extreme heat events have raised concerns about their cascading impacts on health, livelihoods, ecosystems, and the economy and have stimulated ongoing discussion on the causes of such heat extremes.

The past two decades in particular have seen a number of record-shattering summer heat extremes across the Northern Hemisphere mid-latitudes, including the European heat wave of 2003 (3, 4), the Russian heat wave in 2010 (5, 6), and the Texas heat wave and drought in 2011 (7). Importantly, each of these events was directly influenced by quasi-resonant planetary wave amplification or “QRA” (8–10). QRA favors highly persistent summer weather extremes through the resonance of quasi-stationary planetary-scale Rossby waves with their free synoptic-scale counterparts. Resonance generates unusually high amplitudes in higher wave numbers, as quasi-stationary planetary waves with zonal wave numbers 6 to 8 become effectively trapped within midlatitude waveguides of quasi-stationary free synoptic-scale waves, a response that is usually weak under normal atmospheric conditions. Recent work suggests that this phenomenon, which is not well-captured in current-generation climate models due to limited confidence in the wave dynamic response to climate change (11, 12), is becoming more prevalent as a result of Arctic amplification associated with anthropogenic greenhouse forcing (12, 13).

Arguably the most profound and unlikely of recent heat extremes was the now-infamous Pacific Northwest (PNW) “Heat Dome” event in June 2021 (14) with temperatures exceeding 116°F (47 °C) in Portland, Oregon, and 107°F (42 °C) in Seattle, Washington, and the remarkable duration spanning from late June into early July. The temperature extremes during the PNW heat anomaly were so uniquely anomalous that it is difficult to use conventional non-stationary extreme value methods applied to the observational record to characterize the likelihood of the event, even accounting for climate change (14, 15). Evaluation of large ensembles of climate models suggests temperature anomalies exceeding a 4.5 times SD (σ) from the ensemble’s mean at weather stations to be a virtually impossible event (14, 16) in the absence of human-caused warming (while we express the departure from the mean in terms of the SD, σ, this measure of event rarity should not be interpreted in terms of probability derived from Gaussian distributions). Event attribution analyses find that climate change caused the event to be at least 1 to 2 °C warmer, but definitive estimations of its true rarity are elusive (14, 15). It is clear that such a temperature anomaly is very rare and raises the question of whether there are other processes involved that are not properly resolved by current generation model simulations that form the basis of these attribution exercises (17).

**Fig. 1.**
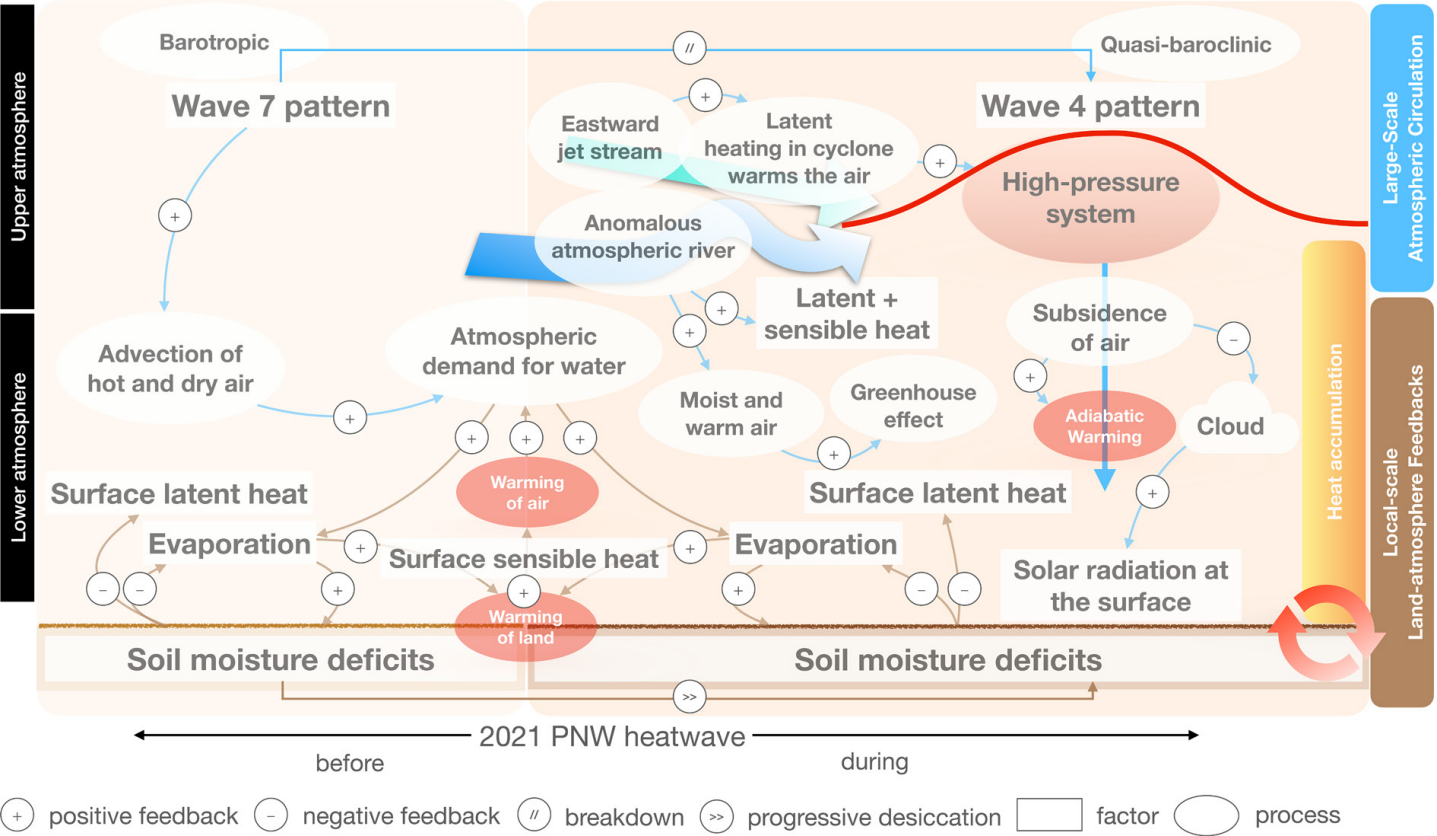
Schematic illustration of the confluence of factors, including large-scale atmospheric circulation and local-scale land-atmosphere feedbacks, underlying the 2021 Pacific Northwest heat-wave event.

Understanding the physical drivers and mechanisms behind the 2021 PNW heat wave requires both a thermodynamic and dynamical perspective. It has been hypothesized that this mega heat wave was largely enabled by the persistence of large-scale dynamics and significantly exacerbated by thermodynamic processes (18). In light of this, several mechanisms have been proposed and are visualized in a conceptual diagram (Fig. 1). This episode has generally been attributed to an upper-level high-pressure atmospheric system (also known as heat dome) (19) in the form of an “Omega Block”. Such blocking anticyclones are associated with adiabatic warming from subsidence of air, which inhibits cloud formation, enhances solar radiation at the surface (20), and reinforces diabatic heating through surface sensible heat fluxes (21, 22). The amplified upper-level ridge in this case was predominantly of tropical origin, involving a rare anomalous North Pacific atmospheric river a few days prior transporting moisture from Southeast Asia and injecting sensible heat energy into western Canada (23) channeled by an extratropical Rossby wave train which, in turn, was triggered by the Southeast Asian summer monsoon anomaly (24, 25). Under the influence of this strong anomalous high-pressure system, the buildup of unsaturated water vapor may have initiated a positive feedback, intensifying the heat wave (23).

Another possible contributor to the event was upwind latent heating south of Alaska, which provided a significant source of downwind wave activity flux, which then converged over western Canada to form the Omega Block (26, 27). Heat waves can further be intensified by local land–atmosphere feedbacks via surface sensible heat from dry soils (28, 29) and advected sensible heat from upwind climatologically warmer regions (30). Such preconditioning was present in most of Oregon and part of Washington State in June during the onset of the PNW heat wave (28).

In any case, it remains unclear why the magnitude of the PNW heat wave—a > 4σ event—was as large as it was, given that the high-pressure system that was in place (the geopotential anomaly) was only a ~3σ anomaly. Here, we seek a better understanding of the underlying physical mechanisms that might explain the uniquely extreme nature of the PNW heat wave. One of the key mechanisms that here-to-fore has received little attention is the role of a QRA event that took place in the weeks leading up to the heat wave. During the first half of June 2021, we witnessed a remarkable hemisphere-wide array of extreme weather events tied to the resonant amplification event. Among them was the record heat in the central and eastern United States with triple-digit heat indices measured in Fahrenheit (>37 °C) covering large swaths of the region and a third of the American population subjected to dangerous heat. Record flooding in Montana impacted Yellowstone National Park, aided by early snowmelt—an example of the increasingly common phenomenon of a concurrent extreme weather event. In France, all-time heat records fell in many locations in early June, nearly two months before typical peak summer heat. Northern Italy suffered from excessive heat and drought.

In this study, we show that this QRA event played a critical, indirect role in the PNW heat wave. We show that the extreme warmth was a result of both the atmospheric circulation state (i.e., Omega Block/ridge) that was in place at the time of the heat wave, and the anomalous low soil moisture and associated land surface feedbacks that resulted from a persistent antecedent atmospheric state associated with QRA which favored advection of dry, warm air into the region over a more than two-week long period. Other potential antecedent conditions, such as the loss of spring snow cover, may likewise contribute to amplified warming through earlier depletion of soil moisture and enforcing high-pressure ridging through a stationary Rossby wave response (31). It is important to note that these factors, while significant, are not within the scope of the present study.

## Results

**Fig. 2.**
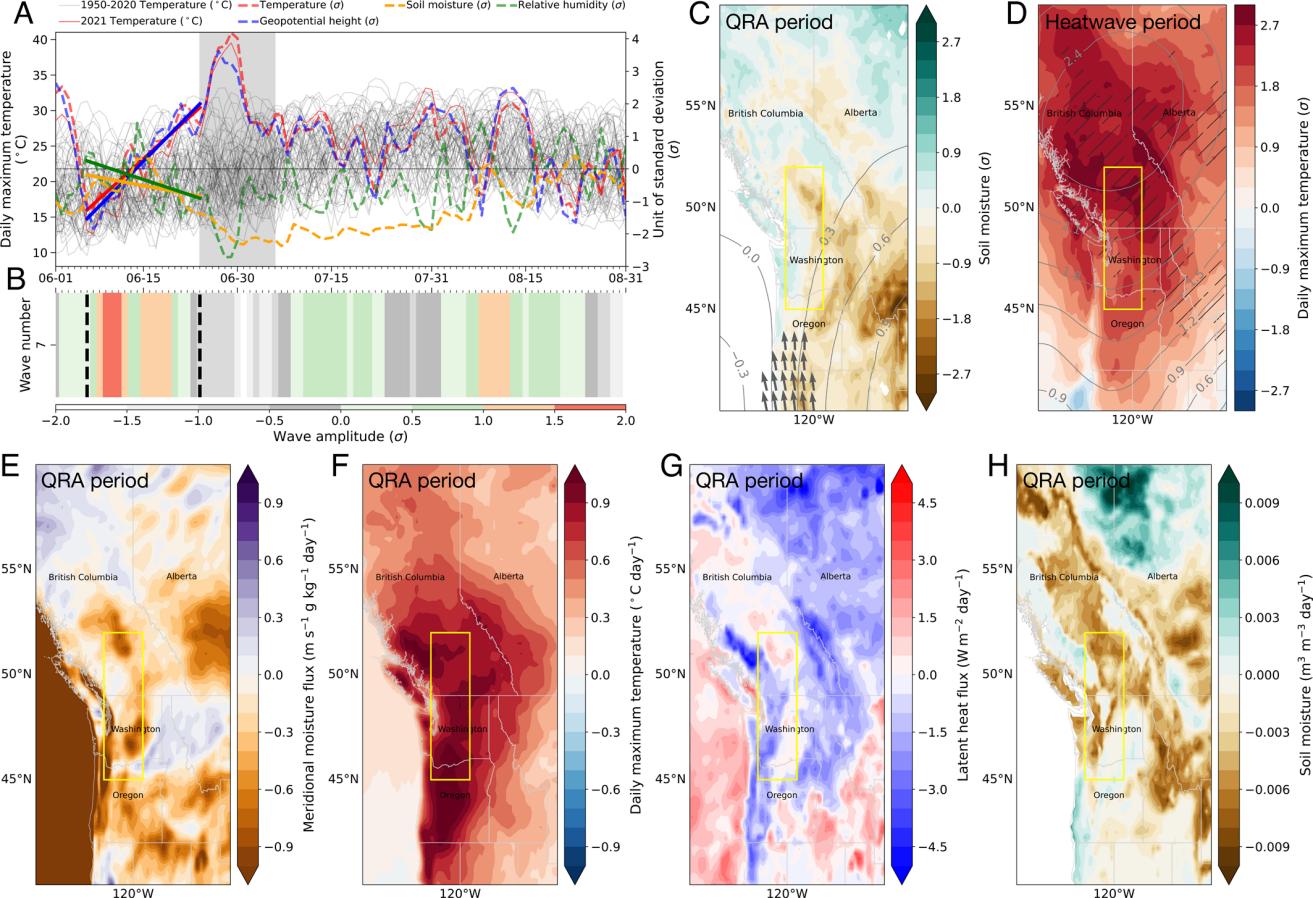
Persistent wave 7 circulation pattern before the onset of the 2021 Pacific Northwest heat wave. (*A*) Evolution of anomalies (dashed colored lines) in area-weighted average of daily maximum temperature (Tx), geopotential height at 500 hPa (Z500), surface soil moisture (SWVL1, 0 to 7 cm soil layer 1), and relative humidity (RH) at 1,000 hPa during Northern Hemisphere (NH) summers over the Pacific Northwest (PNW) land region (45° to 52°N, 119° to 123°W, with ocean masked out from the domain). The anomalies are measured in units of SDs (σ) from the climatological mean of the preceding decades (1950 to 2020). The thin black horizontal line indicates where σ equals zero. The thin red line represents 2021 summer Tx and the thin black lines represent Tx values for individual years. The trend lines for these variables, observed when the wave 7 pattern persists, are indicated by thick colored lines. These trends are statistically significant at the 90% confidence level. For reference, a full set of the variables analyzed can be found in *SI Appendix*, Fig. S1. The record-breaking PNW heat wave from June 24 to July 6, 2021 is highlighted in gray box. (*B*) The detected quasi-resonant amplification (QRA) period for wave 7. The wave amplitude of the mid-NH meridional wind (37.5° to 57.5°N) is displayed in shading, expressed in units of σ. The start and end day of resonance is marked with dashed black lines. (*C*) Spatial patterns of anomalies during the QRA period (see dashed black lines in *B*). (*D*) Same as (*C*) but for the heat wave period (see gray box in *A*). Anomalies in SWVL1 (Tx) and Z500 are indicated by the brown to green (blue to red) shading and gray contour, respectively. Anomalies of 300 hPa wind vectors larger than 1σ are displayed by gray arrows in (*C*). Regions where surface soil moisture anomalies are larger than 1.5σ are hatched in black in (*D*). The corresponding spatial pattern for the mid-NH is provided in *SI Appendix*, Figs. S4 and S5. (*E–H*) Trends in anomalies during the QRA period for (*E*) meridional moisture flux, (*F*) daily maximum temperature, (*G*) surface latent heat flux, and (*H*) surface soil moisture. The yellow box in (*C*-*H*) indicates the PNW region.

For the purpose of this study, we focus on the region of Oregon and Washington in the Pacific Northwest of the continental U.S. that was most impacted by the heat wave, but the results are not impacted by the specific choice of region (*SI Appendix*, Fig. S1 and Table S1). We follow the evolution of the event from its origins at the beginning of June, when a burst of heat generated upwind of the PNW region contributed to an initial temperature peak (Fig. 2*A*; see also *SI Appendix*, Fig. S2). After this “pre-heat wave”, temperature anomalies increased steadily throughout June and culminated in the record-breaking values on 29 June, when the anomaly in daily maximum temperature (Tx) was observed to be 4.2σ from the climatological mean. In fact, the anomalous nature of the event was even more pronounced in the summertime maximum daily maximum temperature (as high as 4.7σ relative to the 1950 to 2020 mean; see *SI Appendix*, Fig. S3), as noted previously (32). The maximum anomaly in geopotential height at 500 hPa (Z500) is roughly coincident with the maximum anomaly in the Tx profile. However, Tx plateaued several days after the anticyclone (as measured by Z500) reached its maximum intensity. While the former was an extreme, over 4σ outlier (note that only five other heat waves were found to be more extreme globally since 1960 when applying the same extreme index calculation described in Thompson et al. (33)), the latter was only a 3.6σ anomaly. That suggests that mechanisms other than the concomitant atmospheric circulation anomaly must have played a critical role. The role of anomalous pre-existing soil moisture deficit is evident from the monotonic drop in surface soil moisture from 15 June onward.

To unravel potential pathways to the anomalous drying, we examine the state of the upper atmosphere during early and mid-June, during which we detect an anomalously high-amplitude resonant planetary wave 7 pattern about two weeks before the onset of the pronounced late June/early July heat wave (Fig. 2*B*). There is a substantial negative trend in relative humidity anomaly associated with the resonant planetary wave over the duration of the interval (Fig. 2 *A* and *B*), consistent with a prevailing atmospheric circulation anomaly that favored drying via anomalous advection of dry, warm air into the region due to anomalous southerly continental flow (Fig. 2*C*). This phenomenon is substantiated by the southward transport of moisture, equivalent to a negative transport of moisture deficit, as illustrated in Fig. 2*E*. The sustained wave 7 pattern persists over much of June, contributing to a positive trend in air temperatures (Fig. 2*F*). Higher temperatures increase atmospheric demand for water, resulting in increased evaporation, as indicated by the negative trend in surface latent heat flux (Fig. 2*G*), leading to a negative trend in soil moisture (Fig. 2*H*) and a steady loss of soil moisture (a maximum of −3.3σ prior to the heat wave period and −2.8σ during it). Consequently, the interplay between this extended QRA circulation state and local land–atmosphere coupling leads to a steady ramp up in temperature (and geopotential height) via decreased evaporative cooling and latent heat flux and increased surface sensible heat flux. While these associative relationships are inferred in terms of causal physical connections, future model-based studies would complement our empirical approach. The progressive built-up of heat sets the stage for an unprecedented heat wave wherein an upper-level high-pressure system remains in place thereafter (Fig. 2*D*).

**Fig. 3.**
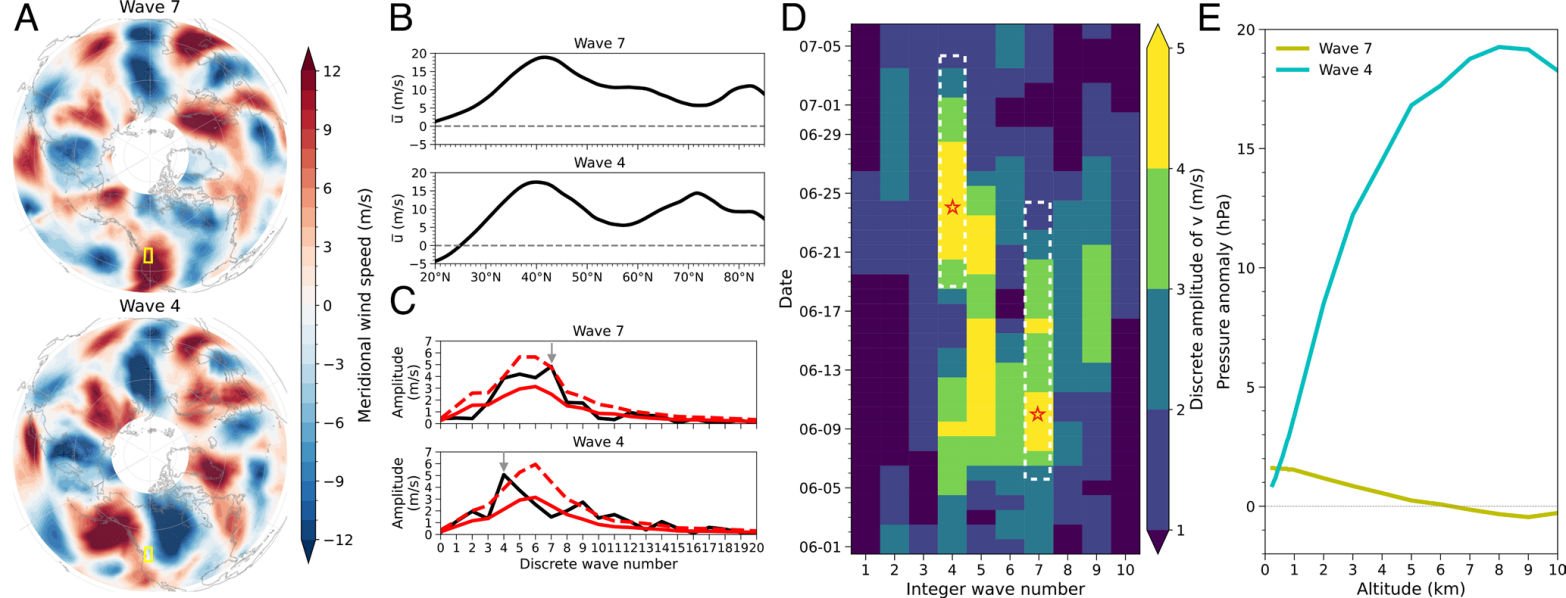
Wave pattern and propagation during June and July of 2021. (*A*) Map of the meridional wind fields at 300 hPa (υ) averaged over the periods when waves 7 and 4 dominate, respectively (see white dashed box in *D*). The yellow box indicates the PNW region. (*B*) Zonal mean zonal wind at 300 hPa ($\bar{u}$) with a double-jet feature. Note that the presence of a double-jet pattern does not always imply summer heat extremes (35). (*C*) Zonal wave number spectra computed by applying a Fourier transform to 15-d running means of υ averaged over 37.5° to 52.5°N (in black) centered on a given date when the amplitude of υ is the highest for each wave pattern (see red star in *D*). For comparison, the climatological mean spectra and the associated 1.5σ upper bound are illustrated in red solid and dashed lines, respectively. Gray arrows indicate the peaks of waves 7 and 4. (*D*) Two-dimensional plot showing discrete amplitude of υ evolved with time and integer wave number. The white dashed box indicates the detected wave 7 pattern during the QRA period and the established wave 4 pattern during the record-shattering heat wave, respectively. (*E*) Pressure anomaly change (hPa) as a function of altitude (km) within the PNW. Note a roughly barotropic anomalous structure (small change in pressure anomaly with height) for the wave 7 patterns and a markedly baroclinic structure (large variation in pressure anomaly with height) for the wave 4 pattern.

A close investigation into the nature of the underlying planetary wave dynamics reveals the evolution from a zonal wave number 7 configuration associated with QRA to a non-resonant zonal wave number 4 configuration as the heat wave forms and develops (Fig. 3). The characteristic features of the resonant wave 7 pattern are illustrated by the meridional wind at 300 hPa (Fig. 3*A*), zonal mean zonal wind (Fig. 3*B*), and zonal wave number spectra (Fig. 3*C*). The quasi-resonant patterns with a dominant wave number 7 contribution are prominent before the heat extremes rage (Fig. 3*D*). The vertical structure is roughly barotropic (Fig. 3*E*), consistent with the assumptions of underlying quasi-resonant amplification. The wave 7 pattern eventually breaks down in late June, evolving instead into a highly baroclinic wave 4 pattern (Fig. 3 *D* and *E*) associated with a deep, anomalous ridge (the Heat Dome). The evolution of the zonal wave number 4 Rossby wave pattern, which is established around 19 June, reaching peak amplitude on 24 June during the heat wave (Fig. 3*D*), is in line with previous findings (28). While a stationary zonal wave number 5 precursor pattern has been identified for US heat waves in some model simulation studies (34), we highlight the remarkable role of a QRA-related zonal wave number 7 in this event, which favored heat transfer and soil moisture–temperature coupling before the heat wave event itself.

## Discussion

Anomalies in both circulation and land-surface processes are necessary to explain the 2021 PNW heat wave event. Here, the impact of the circulation anomaly prior to the event pre-conditioned the atmospheric state in a manner favorable for soil moisture depletion. That in turn created an environment where land–atmosphere interactions could amplify the effects of the anomalous circulation that coincided with the event.

**Fig. 4.**
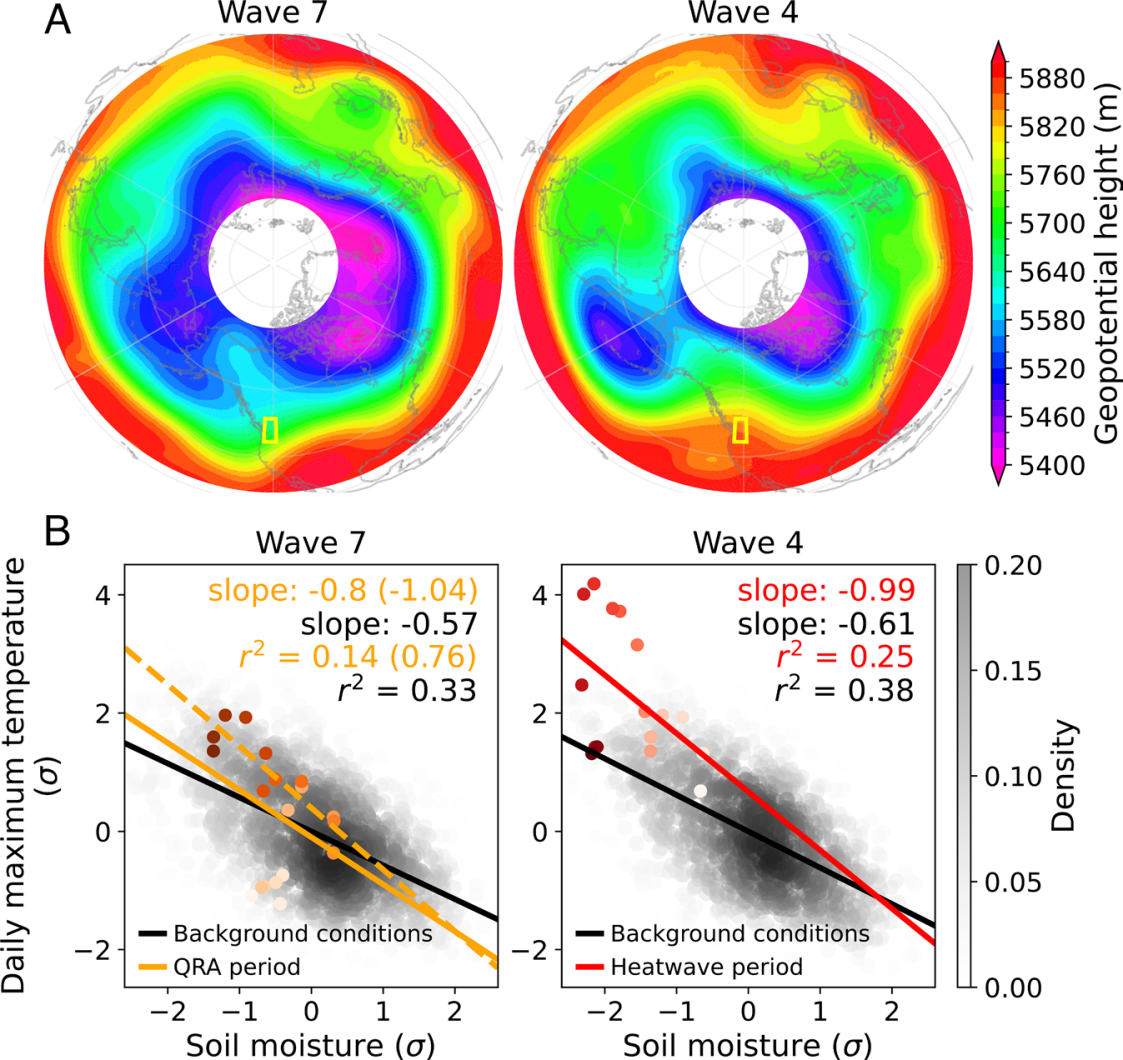
Impact of different wave patterns on record-shattering heat wave at different stages. (*A*) Polar stereographic projection of 500 hPa geopotential height fields (in units of meters; m) averaged over the wave 7 and wave 4 regimes characterized in this study, respectively. The yellow box indicates the PNW region. (*B*) Daily maximum temperature anomalies against soil moisture anomalies during the wave number 7 and wave number 4 regimes, respectively. The density scatterplot compares the relationship for boreal summer (June–July–August) over the reference period of 1950 to 2020 (black and white) and for 2021 (colored dots), with darker dots denoting more recent dates. The fitted lines, rate of change (slope), and coefficient of determination ($r^{2}$) are the results of linear regression performed on the respective dataset for background conditions (in black), QRA period (in orange), and heat wave period (in red). While the solid orange line indicates the full QRA period when wave number 7 dominates in 2021, the dashed orange line represents the QRA period from mid-June onward, with the associated statistics shown in brackets.

Rather than emphasizing the direct role of QRA during heat wave events through phase locking and amplified individual quasi-stationary high-pressure systems (36, 37), this investigation reveals how QRA events may affect antecedent atmospheric conditions (in this case, through land-surface processes), introducing an additional, though indirect, mechanism through which QRA impacts extreme heat events. While the planetary wave activity, as inferred from geopotential height at 500 hPa, exhibits fundamentally different character between the wave 7 pattern that preceded the event and the wave 4 pattern that occurred with it, both significantly impacted the PNW heat wave event in synergistically different ways (Fig. 4*A*). Note that the background climate anomalies do not change too much before and during the event (Fig. 4*B* and *SI Appendix*, Fig. S6). Nevertheless, as the upper-air circulation shifts toward the QRA, local soil moisture–temperature coupling is progressively strengthened, particularly after mid-June, as evidenced by the strong correlation between daily maximum temperature and soil moisture anomalies (Fig. 4*B*). Indeed, the PNW experienced an extended period of rainfall deficit from mid-June onwards, although there were restricted episodes of rainfall in the southwestern portion of the domain on June 5 to 7 and June 13 to 15. It is believed that in situations when wet conditions initially prevail, the frequent presence of anticyclonic regimes has a limited impact on increasing temperatures enough. However, after a rainfall deficit, temperatures become highly sensitive to atmospheric circulation patterns (38). The reinforced soil moisture–temperature interactions may weaken the constraints that cap maximum summer temperatures (39) and result in record-shattering extremes.

The evolution of the 2021 PNW heat wave, as established in this study, is summarized in Fig. 1. First, in the lower troposphere, a wave 7 pattern advects warm dry air to the PNW region in association with southernly continental flow. That enhances the atmospheric demand for water, favorable for evaporation and intensified soil desiccation (40) when the ridge is on top of it (41). It is this confluence of antecedent and concurrent factors that led to the record-shattering heat. Then, the increased geopotential height and flat mean sea level pressure (not shown) indicate a transition from barotropic to quasi-baroclinic instability that eventually break down a wave 7 to a wave 4 pattern. The record-breaking surface air temperature anomaly is the result of both adiabatic compression and subsequent warming associated with subsidence under the ridge and the deficit of soil moisture accelerated by the QRA, which limits the total energy allocated to latent heat flux, and consequently, more energy becomes available for sensible heating, intensifying surface warming.

The implications of our study are several fold. First of all, we illustrate the subtle manner in which antecedent and concurrent atmospheric states can combine to yield a heat anomaly that would be difficult to explain by either in isolation. This complexity emerges for several reasons: a) the antecedent state, in our example, was favored by resonant planetary wave amplification (QRA), b) previous studies (12, 42, 43) indicate that climate change is leading to an increase in the prevalence of QRA, and c) previous studies (44–46) also indicate that current-generation climate models fail to adequately capture QRA. Collectively, our analysis points to precisely the type of extreme event that current climate model projections likely fail to adequately capture. Neglecting to account for such preconditioning feedback mechanisms could potentially lead to an underestimation of risks associated with future extreme heat projections, particularly in regions where evapotranspiration is constrained by soil moisture availability (38, 47). Our findings confirm and expand upon the prevailing understanding of this unprecedented event, driven by the intricate interplay between large-scale atmospheric dynamics and local land–atmosphere feedbacks (18). Furthermore, we have unveiled the roles of antecedent conditions and remote air masses on extreme events that have been largely overlooked in previous research. Such considerations should be considered when assessing the current and future risk of persistent summer weather extremes based on current-generation model simulations.

Second, it is worth noting the concurrent/cascading emergence of heat waves and wildfires, giving rise to complex compound events that, in turn, exhibit linkages with persistent stationary wave patterns. For example, the presence of a preceding resonant wave 4 pattern, in conjunction with El Niño conditions (and soil moisture anomalies), has been proposed as a contributing factor to the unusually early onset of the 2016 Alberta wildfire (48), where heat extremes may favor a wave 7 pattern (36, 49). On a hemispheric scale, some evidence of synchronized wildfire occurrence has identified a wave 5 to 6 pattern from a climate model large ensemble (50). While our study adds to the body of pathways connecting planetary wave dynamics and extreme weather events, further investigation is required to reconcile insights from observations and models and to disentangle the roles of wave dynamics and land surface feedbacks (e.g., soil moisture deficit, early snowmelt (51), land-cover changes, and land-use practices) in the interplay between wildfires and heat waves.

Finally, our analysis points to a protocol that might be used for more skillful predictions of low-probability high-impact weather extremes that involve compound, spatiotemporally separate factors.

## Materials and Methods

### Reanalysis Data.

We used daily maximum temperatures (denoted as Tx; K), obtained from the European Centre for Medium-Range Weather Forecasts (ECMWF) Reanalysis v5 (ERA5) (52). Due to varying research purposes, the annual maximum daily maximum temperature (TXx; K), which describes the hottest day of the year, is commonly employed to infer health impacts due to extreme heat (53). Our analysis using TXx yielded comparable results, as illustrated in *SI Appendix*, Fig. S3. Additionally, other variables used in our analysis were also provided by ERA5, including geopotential at 500 hPa (m^2^ s^−2^), surface soil moisture (SWVL1, 0 to 7 cm soil layer 1; m^3^ m^−3^), surface latent heat flux (J m^-2^), relative humidity at 1,000 hPa (%), specific humidity at 1,000 hPa (kg kg^−1^), meridional wind at 1,000 hPa (m s^−1^), meridional wind at 300 hPa (m s^−1^), and zonal wind at 300 hPa (m s^−1^). The meridional moisture flux can be calculated using the specific humidity and meridional wind field at 1,000 hPa. All these daily aggregates were averaged from hourly estimates, covering the boreal summer from 1950 to 2021 with a resolution of 0.25° × 0.25°.

To facilitate the detection of quasi-resonant wave amplification (QRA), daily temperature, the meridional and zonal wind fields at 300 hPa were regridded onto a coarser 2.5° grid over the period 1979 to 2021. As ERA5 data do not provide height levels directly, it is interpolated from the pressure level data. For Fig. 3*E*, the geometric height (altitude) on pressure levels was approximated using the formula alt=a×Z/(a-Z) where a represents Earth’s radius, and Z denotes the geopotential height (23 pressure levels ranging from 1,000 to 200 hPa). Subsequently, a linear interpolation method was applied to obtain pressure values at a fixed set of height levels. We computed anomalies in units of SDs (σ) by measuring the divergence from the climatological mean of the preceding decades (1950 to 2020). Area-weighted average of daily values was employed to generate Figs. 2*A*, 3*E* and 4*B*. The results were found to be robust with respect to the size of the domain (*SI Appendix*, Table S1 and Fig. S1), establishing the robustness of our findings.

### Quasiresonant Wave Amplification (QRA).

We employed the detection scheme developed by Kornhuber et al. (9, 54) to identify QRA events. The fundamental theory, as detailed by Petoukhov et al. (10), is summarized below.

In simple terms, free synoptic-scale Rossby waves typically exhibit characteristics of high-amplitude and fast-traveling motion, without any significant external forcing. In contrast, forced large-scale Rossby waves result from prominent large-scale forcings, such as quasi-stationary diabatic and orographic forcings, which arise from land–sea temperature contrasts and topographic features. By nature, for these slow-moving forced large-scale Rossby waves, zonal wave numbers larger than 6 are normally weak. However, when conditions are favorable, e.g., the free synoptic-scale Rossby waves become trapped within midlatitude waveguides, their quasi-stationary component, characterized by zonal wave numbers 6 to 8 (55), can contribute to the persistence and formation of high-amplitude wave structures in forced large-scale Rossby waves thanks to quasi-resonance.

Mathematically, we utilized the linearized quasi-geostrophic barotropic potential vorticity approximation with the Wentzel–Kramers–Brillouin (WKB) method at the equivalent barotropic level (EBL) to describe the forced large-scale Rossby waves:

[1] $$\left(\frac{\partial }{\partial t}+\alpha \frac{\partial }{\partial \lambda }\right)\Delta \Psi^{\prime }+\left(2\Omega -\frac{\Delta -u}{a\;\text{cos}\phi }\right)\frac{\partial \Psi^{\prime }}{\partial \lambda }=V_{T}+V_{O}+V_{F},$$

where t is time;Ψ is the streamfuction at the EBL expressed as a function of latitude *φ* and longitude λ; Ω is Earth’s rotational angular velocity (Ω=2π/(60×60×24)) and a is Earth’s radius (*a* = 6,367,500); α is the atmospheric circulation index, given by $\alpha =\bar{u}/a\;\cos$*φ* where $\bar{u}$ is the zonal mean zonal wind at the EBL; $\text{and}\;V_{T}$, $V_{O}$, and $V_{F}$ in the right-hand side refer to the midlatitude thermal forcing, orographic forcing, and eddy friction, respectively.

For free synoptic-scale Rossby waves, that is, the right-hand side of Eq. **1** equals zero, the wave solutions take the form:

[2] $$\Psi =e^{i(kx+ly-\omega t)},$$

where k and l represent the zonal and meridional wave number, respectively; ω is the frequency of zonally propagating waves.

For the quasi-stationary component (ω≈0) of free synoptic-scale Rossby waves, we have the WKB solution, given as:

[3] $$l^{2}=\frac{2\Omega cos^{3}\phi }{a\bar{u}}-\frac{cos^{2}\phi }{a^{2}-\bar{u}}\frac{d^{2}\bar{u}}{d\phi^{2}}+\frac{sin\phi cos\phi }{a^{2}-\bar{u}}\frac{d\bar{u}}{d\phi }+\frac{1}{a^{2}}-{\left(\frac{k}{a}\right)}^{2}$$

For any given k, $l^{2}$ is a function of $\overset{-}{u}$. The formation of a waveguide relies on $l^{2}$. Since l can be either a real or imaginary number, $l^{2}$, therefore, can change the direction of the wave motion at latitudes where the wave energy is reflected back toward the center of the waveguide and thus prevented from dispersion to higher or lower latitudes. These latitudinal positions are called turning points (TPs). Occasionally, two TPs are observed, approximately at 30°N and 45°N, forming a midlatitude waveguide that traps waves with zonal wave number k≈6-8. This trapping leads to the formation of a double jet pattern with strengthened westerlies in subtropical and subpolar latitudes (37) and weakened westerlies in midlatitude (Fig. 3*B*). The propagation of waves within the waveguide is characterized by $l^{2}>0$ and $\bar{u}>0$ within the TPs, and $l^{2}<0$ and $\overset{-}{u}>0$ outside the TPs. The width of the waveguide is confined between the two midlatitude TPs.

To satisfy the assumptions required for the WKB approximation (i.e., slowly varying amplitude and a rapidly oscillating phase) (56, 57), certain criteria were applied to constrain the shape and position of the waveguide (9, 10). These criteria are as follows: first, the change in the meridional wavelength over latitudes within the waveguide’s interior should be small (|*d*/–1/ad*φ*| < 1). This condition is achieved when the maximum value of $l^{2}$ falls within the range of $l_{\min}^{2}=10^{-13}m^{2}$ and $l_{\max}^{2}=10^{-12}m^{2}$. Second, the total width of the waveguide $W_{k}$ should exceed the characteristic scale of the relevant Airy function ($W_{k}\geq 2^{\circ }$). Third, when two waveguides are present, their distance should be no less than 5° to ensure a complete reflection of waves.

When a quasi-stationary free synoptic-scale Rossby wave k≈6-8 is efficiently trapped in the midlatitude waveguide, the forced large-scale Rossby waves with zonal wave number m of 6, 7, and 8 become much stronger in amplitude ${\tilde{A}}_{m}$, expressed as,

[4] $${\tilde{A}}_{m}=\frac{{\tilde{A}}_{\mathrm{eff}}}{\sqrt{{\left[{\left(k/a\right)}^{2}-{\left(m/a\right)}^{2}\right]}^{2}+{\left(L/a^{2}+R^{2}/L\right)}^{2}{\left(m/a\right)}^{2}}}$$

where L and R are the characteristic Rossby radius and Rossby number for the eddies contributing effectively to the atmospheric near-surface and internal “eddy function”; ${\tilde{A}}_{\mathrm{eff}}$ is the effective forcing amplitude and can be determined by applying a zonal fast Fourier transformation (FFT) to the area-weighted meridional average of the effective forcing.

To validate the calculated wave amplitude, an observed amplitude was determined by applying a FFT analysis to the area-weighted meridional mean of meridional wind over 37.5° to 57.5°N. The observed amplitude was considered to match the calculated amplitude with k=m±0.2 (with an amplitude of 1.5 σ above the climatology) and meet the criterion for at least 25% of QRA days.

For the implementation of the formulas, daily temperature, zonal wind, and meridional wind at 300 hPa on a 2.5° × 2.5° grid from 1979 to 2021 were utilized. To filter out fast-moving free synoptic-scale Rossby waves and retain quasi-stationary planetary-scale Rossby waves, a 15-d running was applied to these variables, following the methodology by Petoukhov et al. (58). This particular window size was found to be the most meaningful and computationally efficient among other investigated rolling window sizes (i.e., 30-, 20-, 15-, 11-, 7-, 5, and 1-d) and some variations of spectral bandpass filtering.

### Statistical Analysis.

To determine the relative contribution of surface soil moisture/geopotential height anomalies to daily maximum temperature anomalies, we employed a linear least-squares regression analysis. This statistical approach allowed us to establish relationships between these variables and quantify their associations. In our regression analysis, we utilized the coefficient of determination ($r^{2}$), which is the square of the correlation coefficient (r). The $r^{2}$ represents the proportion of the total variance in the dependent variable (daily maximum temperature anomalies) that can be explained by the independent variable (surface soil moisture/geopotential height anomalies). It provides a measure of the goodness of fit of the regression model and indicates the strength of the relationship between the variables.

## Supplementary Material

Appendix 01 (PDF)

## Data Availability

All study data are included in the article and/or *SI Appendix*.
