# Supplementary material for: Role of atmospheric resonance and land–atmosphere feedbacks as a precursor to the June 2021 Pacific Northwest Heat Dome event
Source: Proc Natl Acad Sci U S A. 2024 Jan 16;121(4):e2315330121. doi: 10.1073/pnas.2315330121 (PMC10823217; doi:10.1073/pnas.2315330121)
Supplement: Supplementary file 1 — Appendix 01 (PDF) [file pnas.2315330121.sapp.pdf]

PNAS

Supporting Information for
Role of atmospheric resonance and land–atmosphere feedbacks as a precursor to the June 2021 Pacific Northwest Heat Dome event

Xueke Li[1], Michael E. Mann[1], Michael F. Wehner[2], Stefan Rahmstorf[3,4], Stefan Petri[3], Shannon Christiansen[1], Judit Carrillo[1]

Corresponding authors: Xueke Li and Michael E. Mann
Email: xuekeli@sas.upenn.edu or mmann00@sas.upenn.edu

This PDF file includes:

Figures S1 to S6
Tables S1

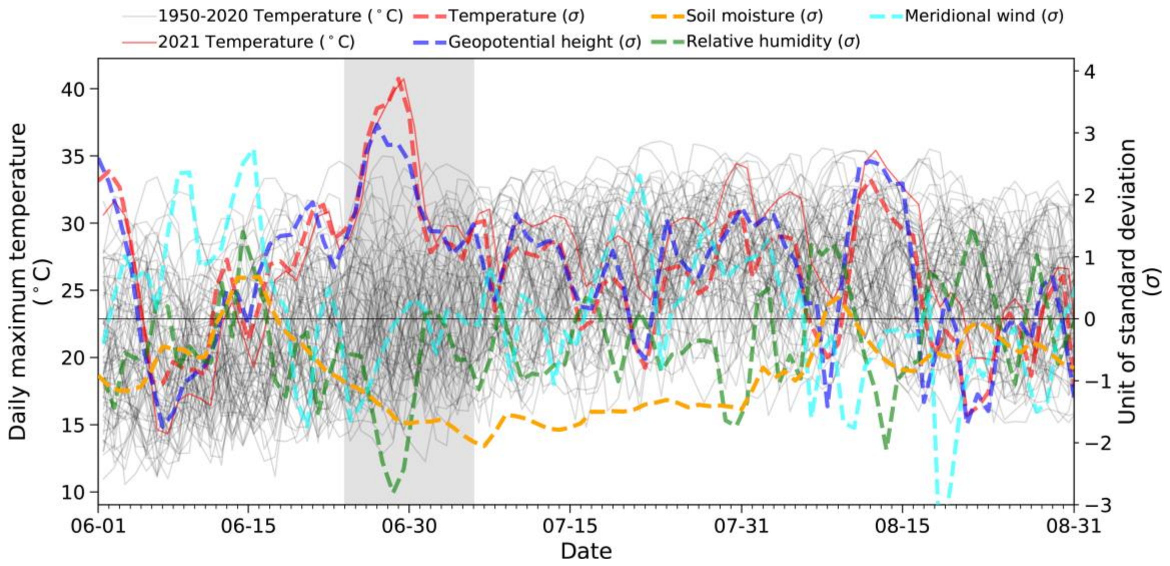

Fig. S1. Same as Fig. 2A in terms of the evolution of anomalies but also includes the meridional wind field and weighted over the Pacific Northwest (PNW) land areas of the U.S. (45°–49°N, 119°–123°W). Note that the heat dome was indeed centered in central British Columbia.

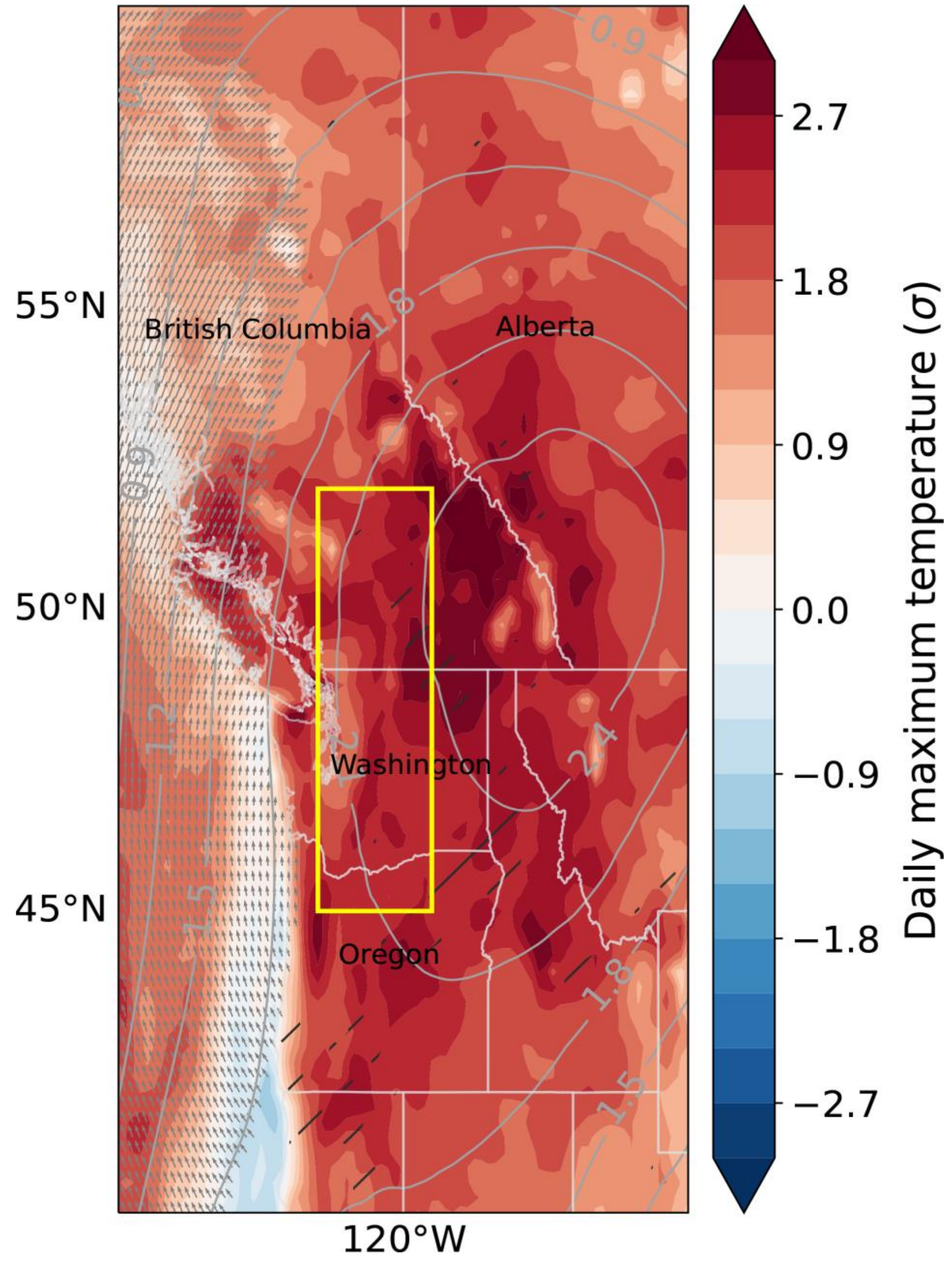

Fig. S2. Spatial patterns of anomalies on 2 June 2021. The anomaly is measured in units of standard deviations (σ) from the climatological mean of the preceding decades (1950–2020). Anomalies in daily maximum temperature and geopotential height at 500 hPa are indicated by shading and gray contours, respectively. Regions where surface soil moisture anomalies larger than 1.5σ are hatched in black. Anomalies of 300 hPa wind vectors larger than 1σ are displayed by gray arrows. The yellow box indicates the Pacific Northwest (PNW) region (45°–52°N, 119°–123°W).

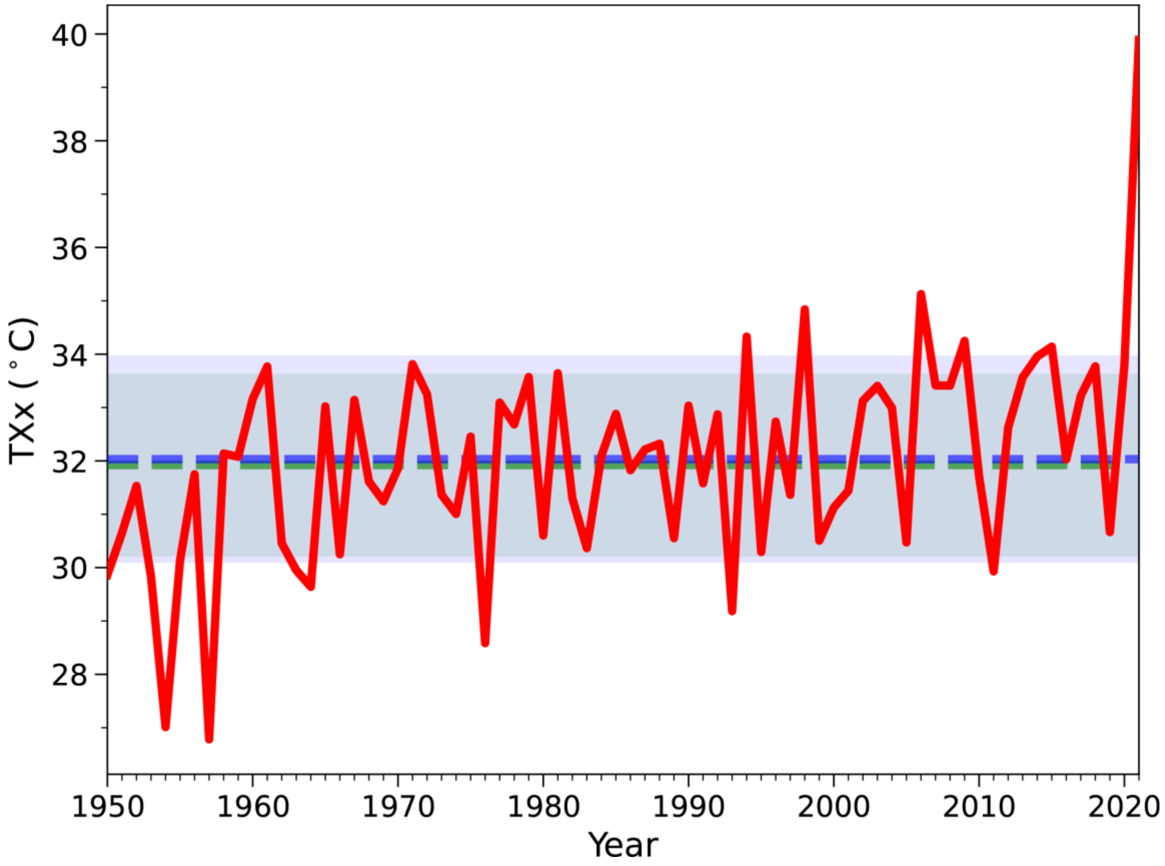

Fig. S3. Time series of the summertime (June, July, and August) maximum daily maximum temperature (TXx) averaged over the PNW region. The historical regional mean (dashed lines) associated with ±1σ uncertainty (shading) is displayed, with blue and green corresponding to calculations with and without the inclusion of the 2021 heatwave event, respectively. The TXx during the 2021 PNW heatwave exhibits exceptional deviations, exceeding 4.7σ and 4.1σ from their respective means for the periods 1950–2020 and 1950–2021.

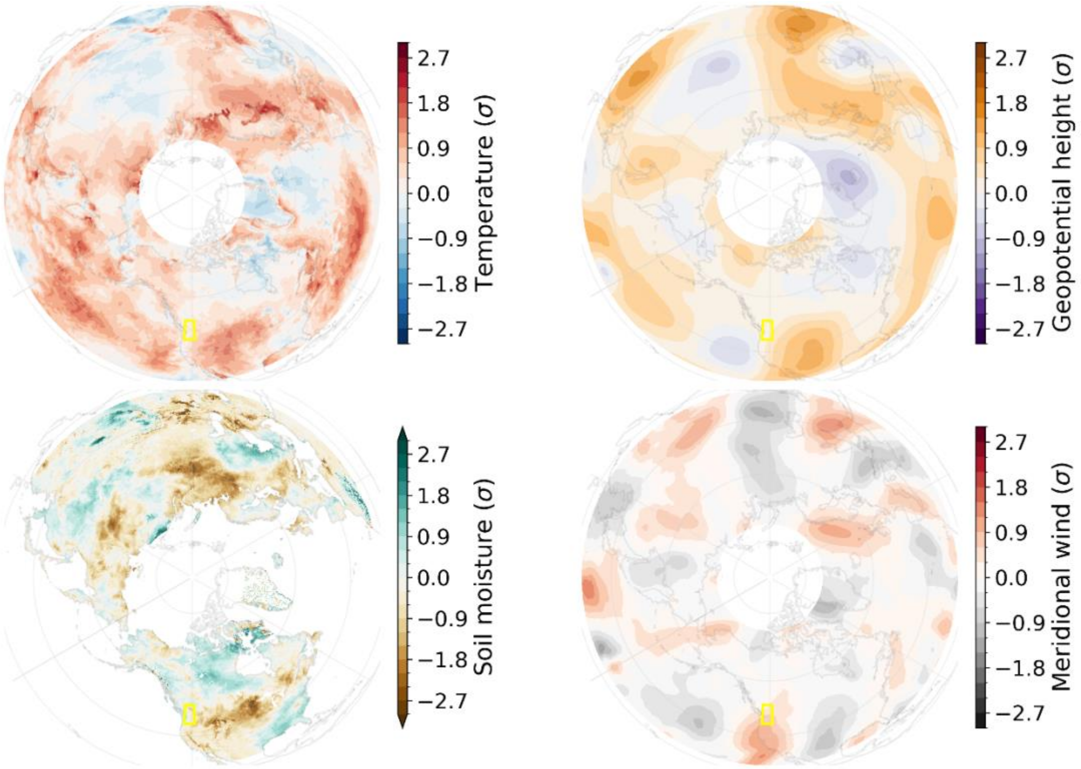

Fig. S4. Spatial patterns of anomalies during the quasi-resonant amplification (QRA) period when a zonal wave number 7 dominates. The yellow box indicates the PNW region.

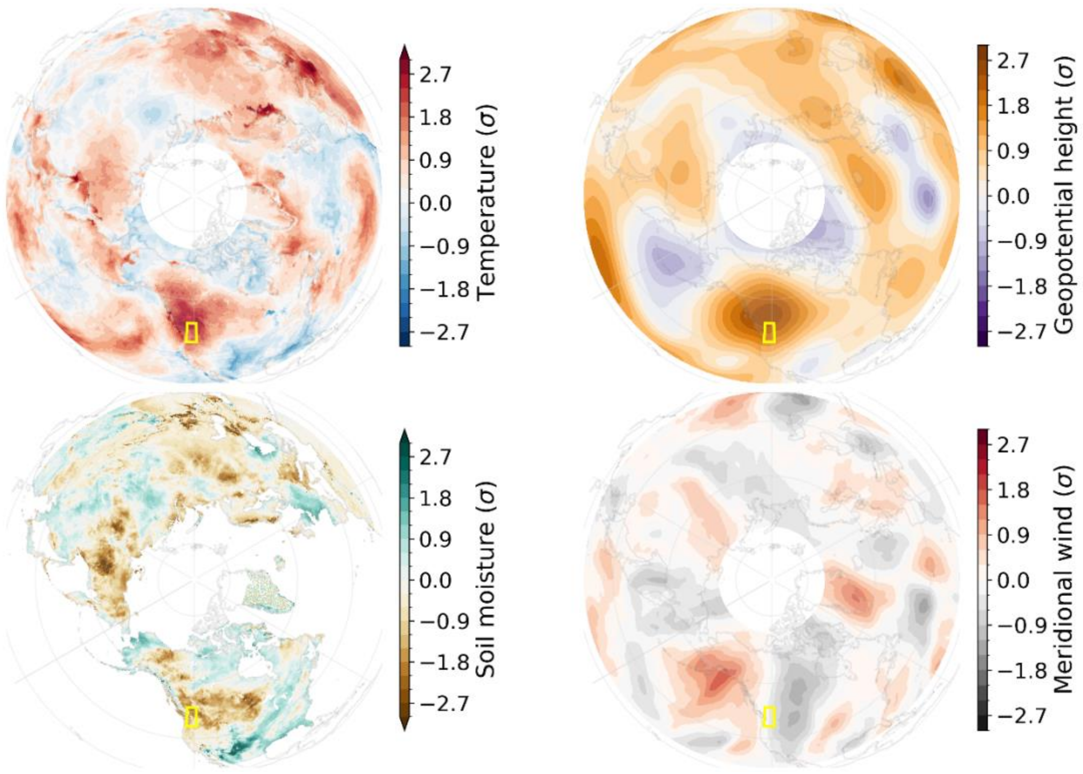

Fig. S5. Same as Fig. S4 but for the heatwave period when a zonal wave number 4 dominates. The yellow box indicates the PNW region.

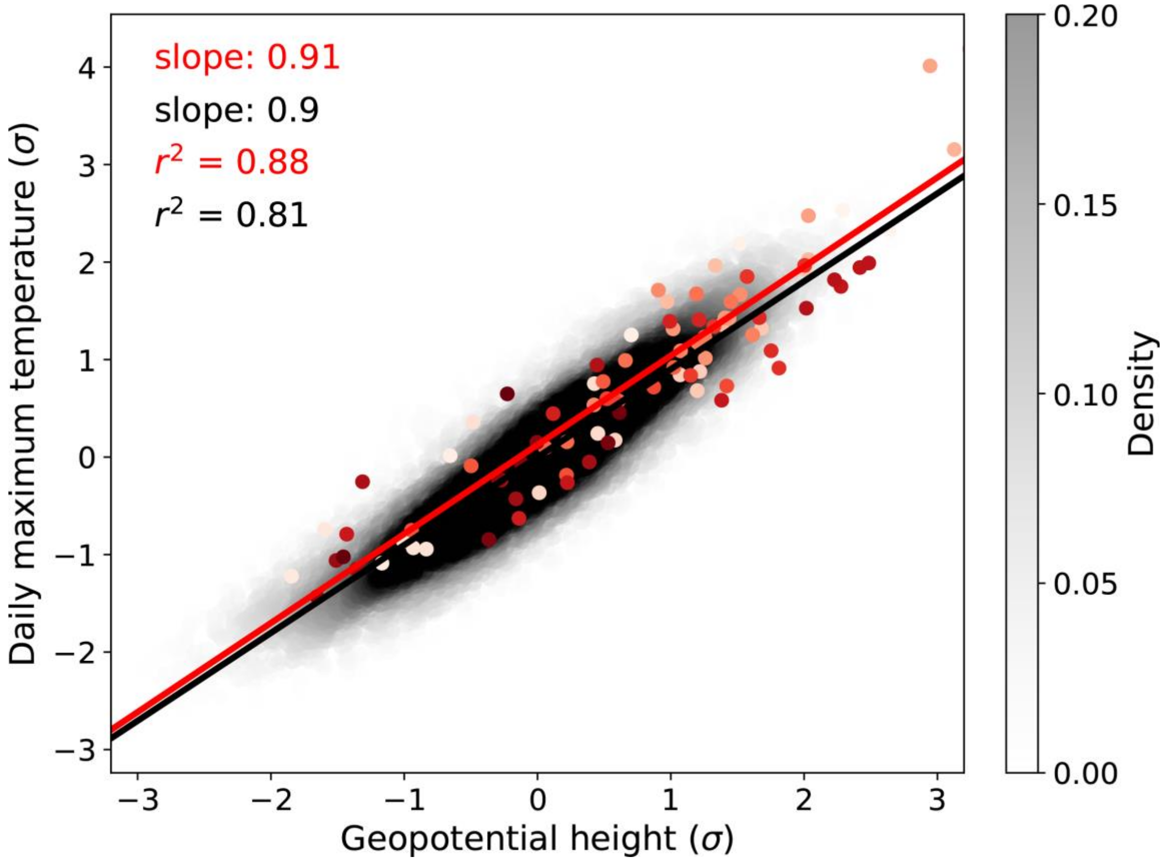

Fig. S6. Daily maximum temperature anomalies against geopotential height anomalies for boreal summer (June–July–August). The density scatterplot compares the relationship over the full period 1950–2020 (black and white) and for 2021 (colored dots), with darker dots denoting more recent dates. The fitted lines, rate of change (slope), and coefficient of determination ($\boldsymbol{r^2}$) are the results of linear regression performed on the respective dataset.

Table S1. Sensitivity of anomalies, measured in units of standard deviations ($\boldsymbol{\sigma}$), to the selection of region when anomalies reach their maximum ($\boldsymbol{\sigma_{max}}$) or minimum ($\boldsymbol{\sigma_{min}}$). PNW is an abbreviation for the Pacific Northwest.

Variable (σ)	PNW land region (45°–52°N, 119°–123°W)	PNW land areas of the U.S. (45°–49°N, 119°–123°W)
Daily maximum temperature (σ_{max})	4.19	3.87
Geopotential height at 500 hPa (σ_{max})	3.62	3.13
Surface soil moisture (σ_{min})	-2.37	-2.05
Relative humidity at 1000 hPa (σ_{min})	-2.72	-2.81
Meridional wind at 300 hPa (σ_{max})	2.83	2.74
